# Identification of New Ketamine Metabolites and Their Detailed Distribution in the Mammalian Brain

**DOI:** 10.1021/acschemneuro.4c00051

**Published:** 2024-03-20

**Authors:** Theodosia Vallianatou, Carina de Souza Anselmo, Ioanna Tsiara, Nicholas B. Bèchet, Iben Lundgaard, Daniel Globisch

**Affiliations:** § Department of Chemistry-BMC, Science for Life Laboratory, Uppsala University, Box 576, 75123 Uppsala, Sweden; † Department of Experimental Medical Science, Lund University, 22362 Lund, Sweden; ‡ Wallenberg Centre for Molecular Medicine, Lund University, 22362 Lund, Sweden

**Keywords:** Ketamine, drug metabolism, brain distribution, mass spectrometry, phase II metabolites

## Abstract

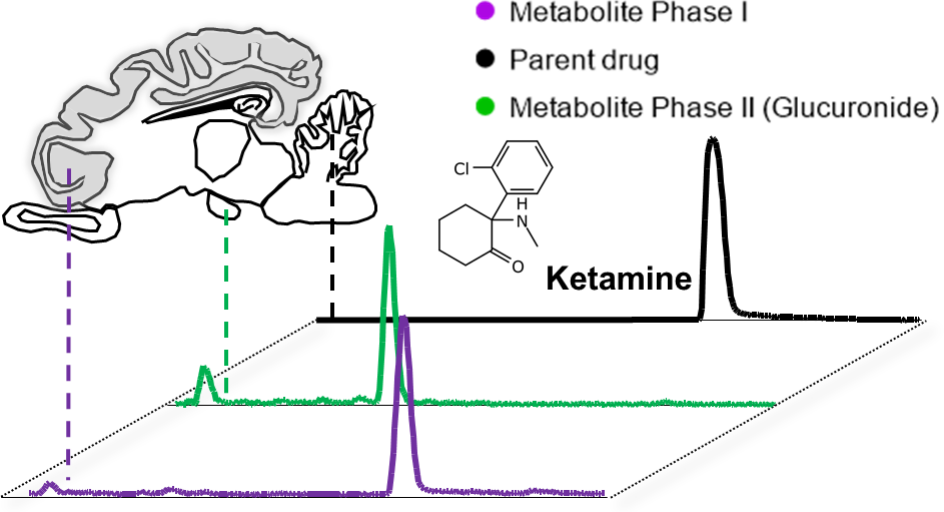

Ketamine is a common anesthetic used in human and veterinary medicine. This drug has recently received increased medical and scientific attention due to its indications for neurological diseases. Despite being applied for decades, ketamine’s entire metabolism and pharmacological profile have not been elucidated yet. Therefore, insights into the metabolism and brain distribution are important toward identification of neurological effects. Herein, we have investigated ketamine and its metabolites in the pig brain, cerebrospinal fluid, and plasma using mass spectrometric and metabolomics analysis. We discovered previously unknown metabolites and validated their chemical structures. Our comprehensive analysis of the brain distribution of ketamine and 30 metabolites describes significant regional differences detected mainly for phase II metabolites. Elevated levels of these metabolites were identified in brain regions linked to clearance through the cerebrospinal fluid. This study provides the foundation for multidisciplinary studies of ketamine metabolism and the elucidation of neurological effects by ketamine.

Ketamine (**KET**) is a synthetic and injectable dissociative anesthetic used for short-term surgical procedures in veterinary and human medicine for decades.^1^ It can be administered either as one of its two enantiomers, *R*- and *S*-**KET**, or as a racemic mixture. Recently, **KET** has attracted more attention due to the high efficiency and rapid effect for the treatment of major depressive disorders, when administered at subanesthetic doses.^2−4^ Furthermore, the misuse of **KET** is an important social problem causing overdose deaths.^5^

Although the anesthetic and analgesic effects of **KET** are considered to be mediated via *N*-methyl-d-aspartate receptor (NMDA) antagonism, a number of different mechanisms have been suggested for its antidepressant pharmacological profile.^1,6,7^ However, the exact pharmacological interactions and the different roles of both isomers have not yet been elucidated. Importantly, a significant amount of evidence suggests that the antidepressant activity of the drug may be mediated by its metabolites.^8−11^**KET** undergoes extensive and stereoselective metabolism mainly in the liver (Figure 1). *N*-demethylation to norketamine (**nKET**) by CYP3A4 was identified as the dominant pathway.^14^**nKET** can be further hydroxylated to **hydroxy-nKET**. Alternatively, and to a lower extent, **KET** is metabolized to hydroxy-KET (**hKET**).^8,12^ A better understanding of **KET**’s distribution and metabolism in the brain is important to shed light into the mechanisms involved in the diverse neuroactive effects of the drug. Nonetheless, only a limited number of studies provide comprehensive insights into the neuro-pharmacokinetics of the drug.^9^

**Figure 1:**
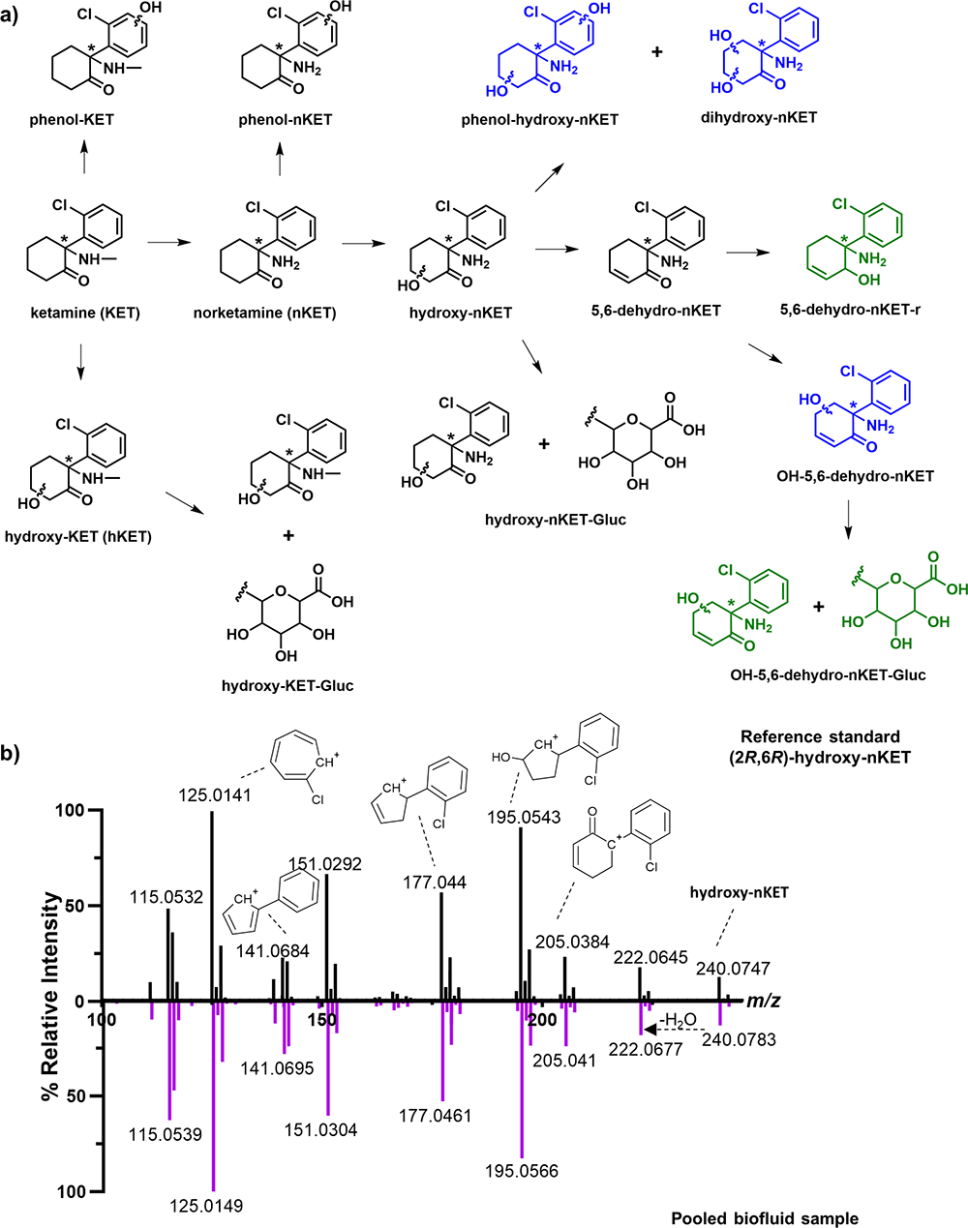
Detection and identification of **KET** and its metabolites. a) Chemical structures of **KET** and its phase I and phase II metabolites detected in the present study. Metabolites illustrated in blue color (**phenol-hydroxy-nKET**, **dihydroxy-nKET**, **OH-5,6-dehydro-nKET**) have previously been reported, but the chemical structure was not identified. Metabolites illustrated in green (**5,6-dehydro-nKET-r**, **OH-5**,**6-dehydro-nKET-Gluc**) have not been previously reported. The asterisk highlights the stereocenter in the **KET** scaffold. b) Structure validation of **(2*****R*****,6*****R*****)-hydroxy-nKET** (*m*/*z* 240.0791) by comparison of tandem spectra collected from the reference standard (black) and a pooled biofluid sample (purple).

In the present study, we have investigated the metabolism and distribution of **KET** in 12 anatomically distinct brain regions isolated from domestic pigs by applying ultraperformance liquid chromatography–mass spectrometry (UPLC-MS) techniques. We have also investigated plasma samples from both the common carotid artery (CCA), which supplies blood to the brain, and the internal jugular vein (IJV), which excretes blood from the brain. These sample types were complemented by analysis of cerebrospinal fluid (CSF). This approach allowed for the comprehensive investigation of the neuro-distribution profile of the drug and the discovery of unknown metabolites. We detected and identified numerous phase I and phase II (glucuronidated) metabolites including previously unidentified metabolites. Furthermore, we found significant differences in the brain distribution, mainly of **KET** phase II metabolites in the regions associated with permeability and clearance. The comprehensive mapping of the distribution of **KET** in the brain reported herein provides new insights into drug metabolism that builds a foundation for future studies in diverse research fields focused on neurological disorders and drug metabolism.

**KET** has a chlorophenyl scaffold that is substituted with 2-methylamino cyclohexanone and its major metabolites have been identified (Figure 1a).^13,14^ Mass spectrometry (MS) is the method of choice for their analysis, and characteristic MS fragmentation profiles were reported for its known metabolites. For simplification of the metabolite structure presentation, we have drawn all metabolite structures without stereochemistry. The stereocenter is highlighted with an asterisk (Figure 1a). Structural elucidation of the stereochemistry of each metabolite would require advanced analysis, such as derivatization methodologies, which would exceed the scope of this study. Most **KET** analogue fragments maintain the chloro-moiety in their structure simplifying the identification of their specific chloro-pattern in the high-resolution mass spectra.

The highest validation level of detected metabolite structures was obtained through coinjection analysis of a pooled biofluid sample with the commercially available reference compound **(2*****R*****,6*****R*****)-hydroxy-nKET** (Confidence level 1).^15^ Moreover, MS/MS spectra acquired for *m*/*z* = 240.0795 in the reference standard and in the biological sample confirmed the presence of this metabolite in plasma and CSF (Figure 1b). The structures of **KET**, **nKET**, and **5,6-dehydro-nKET** were validated by comparison of the MS/MS acquired with MS/MS spectra from experimental or computational libraries with either HMDB, the software SIRIUS or literature spectra [Confidence levels 2a (library) and 2b (experimental), Figure S1].^15,16^ The new detected metabolite structures were validated either at confidence level 2b or 3 through extensive MS/MS fragmentation analysis (Figure 2, Table S1).^15^ Metabolites with a molecular formula including the chloro-group but without an elucidated MS/MS spectrum were classified as confidence level 4.^15^ Furthermore, fragment ion *m*/*z* 125.0155 (2-chlorophenyl-methylium) is stable and conserved among all of the **KET** metabolites. Through these fragments preserving the mass spectrometric pattern of the chloro-group, all derived metabolites of this anesthetic can be distinguished from the endogenous compounds present in the biological matrix (Figure 1b). No complete metabolite structures were obtained for metabolites **M1** and **M2** (Confidence levels 3 and 4) and have not been included in Figure 1. An overview of all of the **KET** metabolites from this study is reported in Table S1.

**Figure 2:**
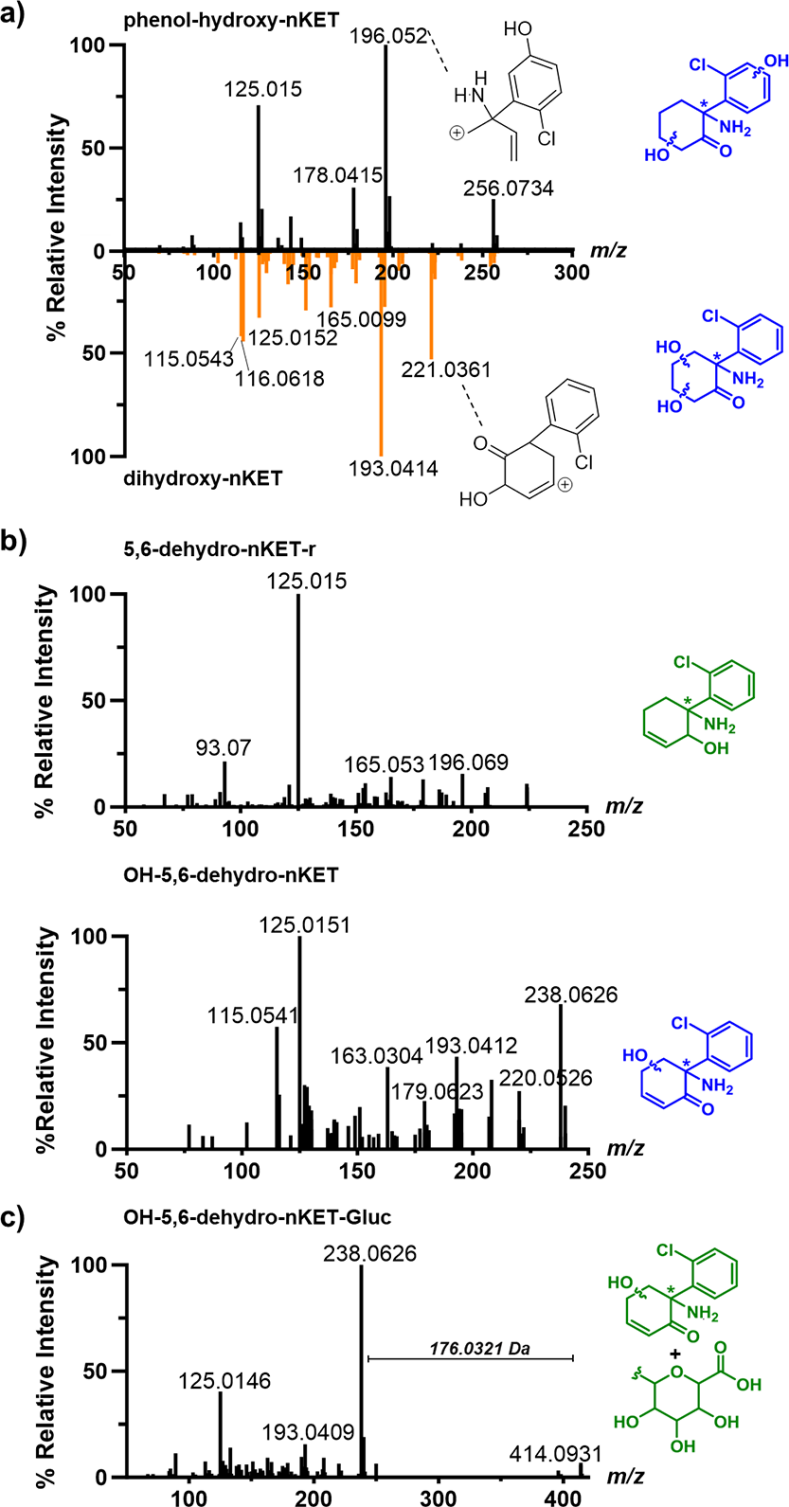
Structure validation of **KET** metabolites. a) Comparison of tandem MS spectra collected in biofluid samples for the isomeric dihydroxylated KET metabolites **phenol-hydroxy-nKET** and **dihydroxy-nKET** (*m*/*z* 256.0741, black and orange spectra, respectively). b) Tandem MS spectra collected in biofluid samples for **5,6-dehydro-nKET-r** (*m*/*z* 224.0842) and **OH-5,6-dehydro-nKET** (*m*/*z* 238.0635). c) Tandem MS spectra collected in biofluid samples for **OH-5,6-dehydro-nKET-Gluc** (*m*/*z* 414.0956).

Hydroxylation is a common phase I drug metabolism reaction that has been described for **KET** at both main moieties resulting in either hydroxylated ketamine (**hKET**) or phenolic ketamine (**phenol-KET**) metabolites. It has so far been difficult to distinguish between a hydroxylation in the aliphatic hexanone or the chlorophenolic moiety (Figures 1a, S1). This is also based on previous reports that the common fragments for ketamine are the hydroxylated analogues *m*/*z* 177.044 and 205.0384 (Figure 1b).^14^ To distinguish between these two possible hydroxylation sites, we focused on the two bishydroxylated metabolites (*m*/*z* 256.0741) that were detected previously but their structure has not been elucidated in a study with human microsomes.^14^

We have now determined their structure experimentally as **phenol-hydroxy-nKET** and **dihydroxy-nKET** in the pig brain for the first time (Figure 1a). The structures were identified due to a difference in the fragmentation spectra with fragments *m*/*z* 196.0527 for **phenol-hydroxy-nKET** and *m*/*z* 193.0419 for **dihydroxy-nKET** (Figure 2a). The *m*/*z* 196.0527 fragment contains one nitrogen atom due to its even protonated mass. The nitrogen rule was applied to assign this metabolite as a hydroxyphenol analogue of **hydroxy-nKET**. Furthermore, the 2-chlorophenyl-methylium ion (*m*/*z* 125.0150) is part of the fragmentation pattern that was utilized to identify both metabolites **phenol-hydroxy-nKET** and **dihydroxy-nKET** as ketamine derivatives. In contrast, the MS fragment *m*/*z* 221.0373 in **dihydroxy-nKET** was identified with a cleaved amine and eliminated water due to its odd protonated mass that does not contain a nitrogen atom due to the nitrogen rule. This fragment also contains the chloro-group and leads to fragment *m*/*z* 193.0419 through the loss of CO (Figure 2a). Consequently, metabolite **dihydroxy-nKET** is a dihydroxylated analogue of nKET.

The new metabolite **5,6-dehydro-nKET-r** has the same molecular formula as **nKET** (C_12_H_14_ClNO) but elutes at a different retention time. The fragmentation spectrum of **5,6-dehydro-nKET-r** validated the core structure of this metabolite to be derived from **KET** with a reduced carbonyl compared to precursor metabolite **5,6-dehydro-nKET** (Figures 2b, S1). Metabolite **OH-5,6-dehydro-nKET** is a hydroxylated analogue of metabolite **5,6-dehydro-nKET** but with a different exact protonated mass (*m*/*z* 238.0633) compared to that of **KET** (*m*/*z* 238.0999). This difference was distinguished by high-resolution MS, and both compounds elute at different retention times. The **KET** core structure was again validated by the MS fragment *m*/*z* 125.0151 with a predicted molecular formula of C_12_H_12_ClNO_2_ that was assigned by the software SIRIUS.^16^ Additionally, the three fragments *m*/*z* 163.031, 179.062, and 220.088 have been described in the literature for **KET**.^13,14^ The metabolite structure of **OH-5,6-dehydro-nKET** (*m*/*z* 238.0633) was further fragmented in our MS/MS analysis into *m*/*z* 146.0609 (C_10_H_12_N^+^) and *m*/*z* 193.0429 (C_11_H_10_ClO^+^). These fragments are specific for **hydroxy-5,6-dehydro-nKET** and can clearly be distinguished from the fragmentation of **KET** (Figures 2b, S1).

Phase II metabolites were also detected, originating from the hydroxylated analogues of **KET** and **nKET**. The specific neutral loss of glucuronic acid (176.0321 Da) in the MS/MS analysis confirmed the conjugated metabolites (Figure S2).^17^ A new phase II metabolite (**OH-5,6-dehydro-nKET-Gluc**, *m*/*z* 414.0960) was identified as well, which originated from metabolite **OH-5,6-dehydro-nKET**. The MS fragmentation spectrum was nearly identical to that of **OH-5,6-dehydro-nKET** after neutral loss of a glucuronide (Figure 2c), which further verifies the presence of the newly discovered phase I metabolite **OH-5,6-dehydro-nKET** in the pig brain. We also identified metabolites **M1** and **M2**, which are both phase II modifications of **KET** metabolites due to the chloro-group and the detected neutral loss of glucuronic acid. However, no structure of the core metabolite with a chemical formula of C_18_H_22_ClNO_7_ could be deciphered (*m*/*z* 400.1163, Table S1). To the best of our knowledge, the five metabolites **phenol-hydroxy-nKET**, **dihydroxy-nKET**, **5,6-dehydro-nKET-r**, **OH-5,6-dehydro-nKET**, and **OH-5,6-dehydro-nKET-Gluc** have been described in vivo for the first time.

After detection and identification of the molecular species derived from **KET** administration, their distribution in plasma (CCA and IJV) and CSF was investigated. We initially performed an exploratory overview analysis using principal component analysis (PCA) of the three biofluid types including a set of 31 features (**KET**, phase I **KET** and phase II **KET** metabolites). The scores plot of the two first principal components, PC1 and PC2, displayed a clear difference between the CSF and both plasma samples (IJV and CCA, Figure 3a). Evaluation of the corresponding loadings plot demonstrates that this difference is mainly attributed to the glucuronidated phase II metabolites (Figure 3b). Interestingly, one metabolite that was identified as **hKETa** (*m*/*z* 254.0947, Figure 3b, d) was found to be more abundant in the CSF compared to that in both plasma samples.

**Figure 3:**
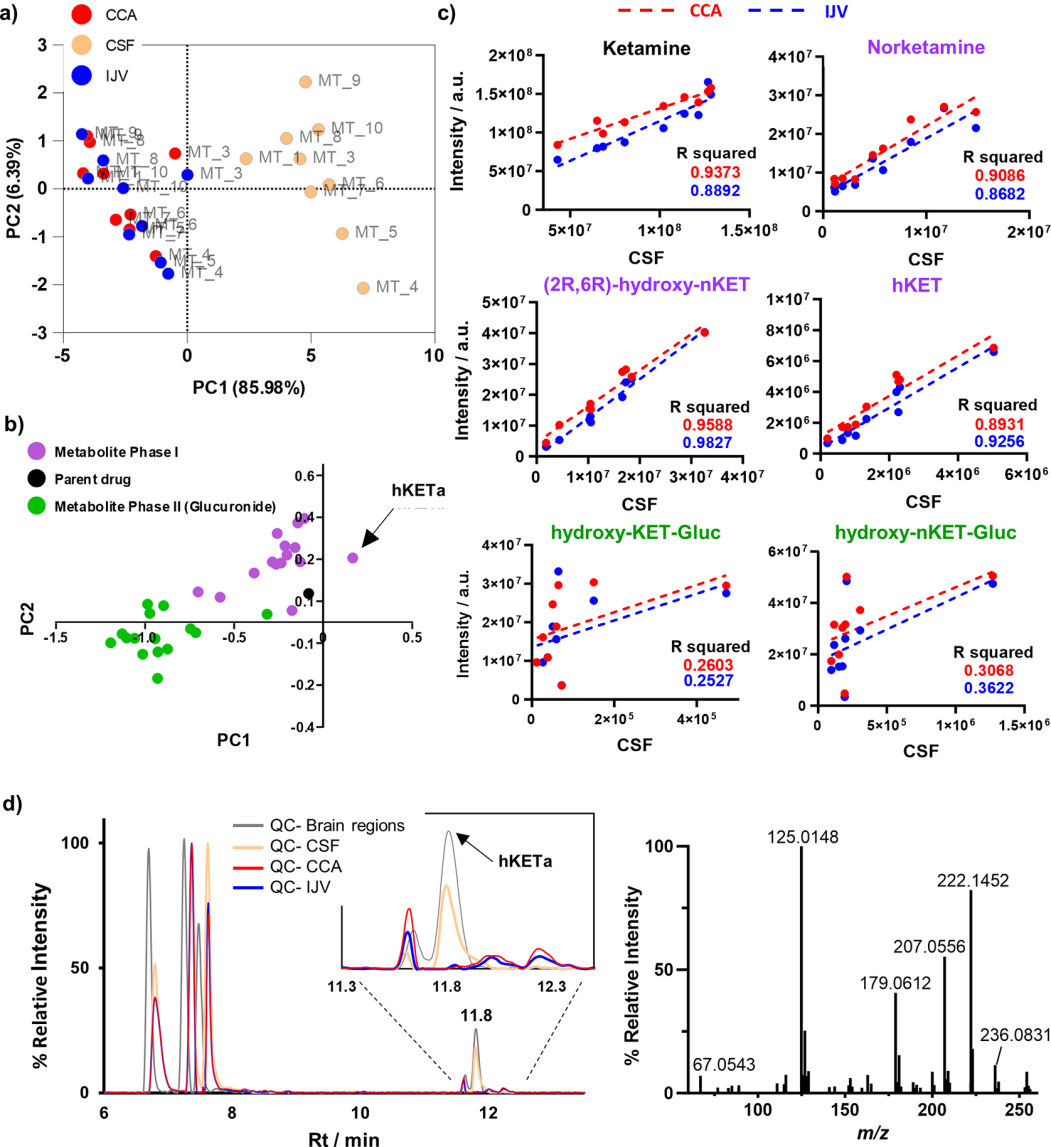
Correlation of **KET** metabolites in plasma (CCA, IJV) and cerebrospinal fluid (CSF). a) Scores plot of the first two principal components from the PCA including **KET** and its detected and identified metabolites. b) Loadings plot of the first two principal components from the PCA including **KET** and its detected and identified metabolites. Black color indicates the parent drug (**KET**), purple color indicates phase I metabolites, and green color indicates phase II metabolites. c) Linear regression analysis for several **KET** metabolites between CCA, IJV, and CSF. d) Overlaid extracted chromatograms from quality control (QC) samples of plasma (QC-CCA, QC-IJV), CSF (QC-CSF) and brain tissue (QC-brain regions) for *m*/*z* 254.0947. The chromatographic peak at 11.8 min corresponds to metabolite **hKETa** (left). Tandem spectra collected in biofluid samples for **hKETa** (*m*/*z* 254.0947) confirm it is a hydroxylated KET derivative (right).

Regression analysis of **KET** and its metabolites between plasma and CSF confirmed that the other phase I metabolites highly correlated between the three biofluids (R^2^ > 0.85). This can possibly be explained as small lipophilic molecules can cross the blood–brain barrier (BBB) and blood-CSF barriers (BCSFB).^9,12^ The glucuronidated metabolites of **KET** and **hKETa** were identified as exceptions (Figure 3c, Table S2). This finding may be associated with differences in the brain permeability and clearance between phase I and phase II metabolites. Importantly, the conjugation of the hydrophilic glucuronic acid moiety to the molecule significantly impacts the entrance into and clearance from the brain for each metabolite as the majority of the mammalian glucuronidation primarily happens in the liver.^18^ Interestingly, phase I metabolite **hKETa** elutes later compared to the other detected hydroxylated KETs and was found to be more abundant in the CSF and brain tissue (Figure 3d). Tandem MS confirmed that **hKETa** is a hydroxylated KET (Figure 3d). Due to the later retention time than the other **hKET** isomers, we conclude that this metabolite is of higher hydrophobicity.

After investigation of KET and its metabolite in the biofluids, the distribution of **KET** and its metabolites was investigated in 12 anatomically distinct regions of the pig brain (Figures 4a, S3). These included cortical regions (frontal, CTX-F; occipital, CTX-O; parietal, CTX-P; temporal, CTX-T), hippocampus (HC), striatum (STRIA), midbrain (MDB), olfactory bulb (OB), cerebellum (CBL), white matter (WM), pituitary gland (PITUI), and choroid plexus (CP). The distribution of **KET** and all 30 detected **KET** metabolites in the brain tissue demonstrated that CP and PITUI were the two brain regions that differed from the other ten as illustrated in the first principal component ([t1]) of the PCA (Figure S3). This is an important finding as CP is a small highly vascularized brain structure located in the brain ventricles that produces the CSF, while PITUI belongs to a group of specialized neuroepithelial structures with more permeable BBB called circumventricular organs.^19−21^ The loadings plot derived from the same analysis revealed an association with the diverse distribution in the brain regions of **KET** and its phase II metabolites (Figure 4b). This was further confirmed through the identification of statistically significant differences in one-way ANOVA as illustrated for four different **KET** metabolites (Figure 4c). While **KET** and **nKET** were distributed unspecifically, their glucuronides were mainly concentrated in CP and PITUI (Figure 4C). The vascularization of these two tissues is more permeable compared to the BBB, which could be an explanation for the accumulation of these hydrophilic glucuronidated **KET** metabolites in CP to excrete metabolites via the CSF. Moreover, UDP-glucuronosyltransferase activity has been specifically detected in CP and PITUI.^22,23^ This can be associated with the high accumulation of the hydrophilic glucuronides in PITUI compared to their lower abundance in other brain regions (Figure 4C).

**Figure 4:**
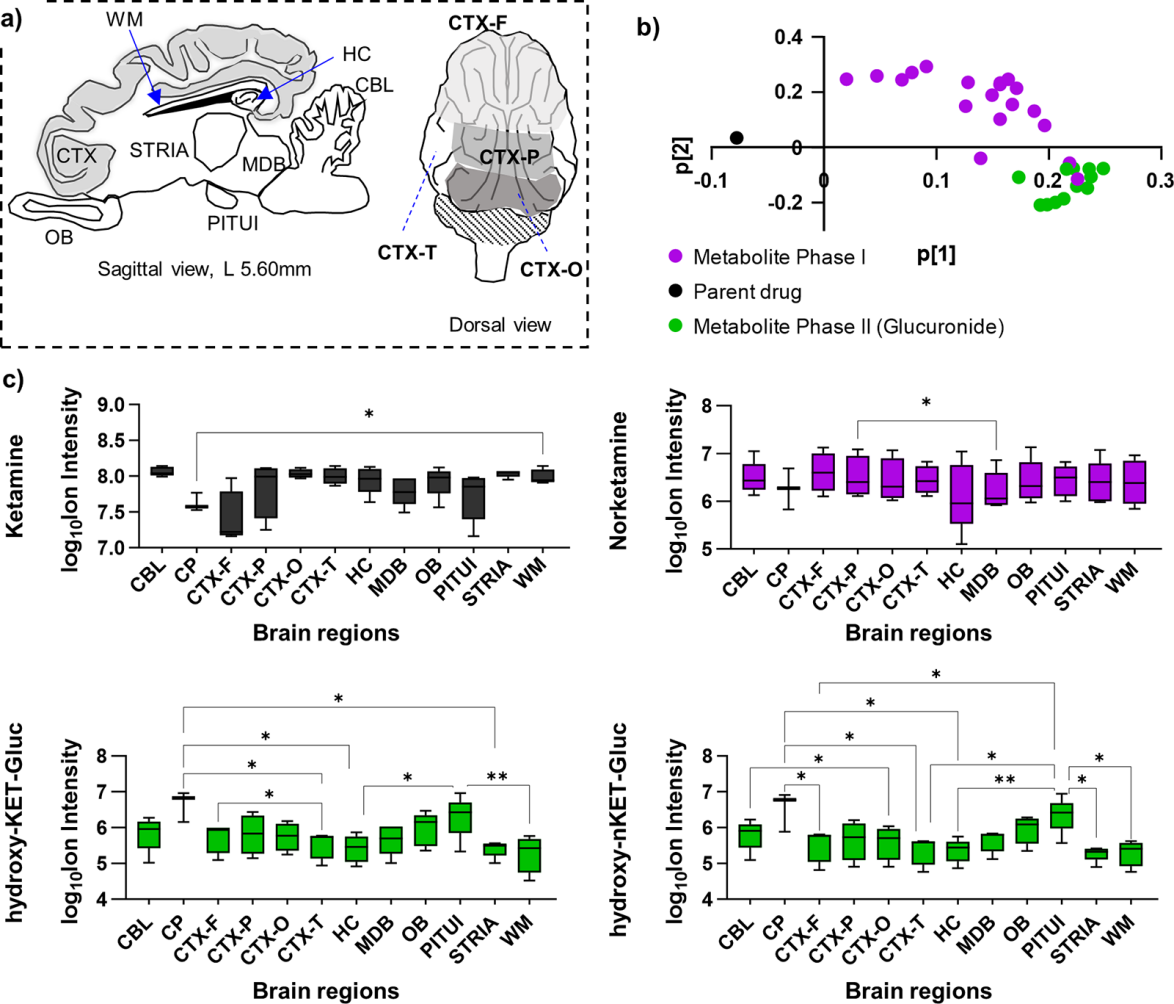
Brain mapping and regional distribution of **KET** and its metabolites. a) Graphical illustration of the pig brain regions investigated in the present study. b) Loadings plot of the first two principal components from the PCA including **KET** and its detected and identified metabolites. Black color highlights the parent drug (**KET**), purple color for phase I metabolites, and green color for phase II metabolites. c) Box plots of brain regional distribution of **KET**, phase I and phase II metabolites. **P < 0.01, *P < 0.05 (one-way mixed-effects ANOVA).

In summary, we herein described the detection of a series of ketamine metabolites in the pig brain and biofluids related to brain entrance and clearance. The distribution of **KET** and its metabolites revealed that phase II modifications are predominantly located in the two brain regions CP and PITUI, which are linked to CSF clearance of metabolites. Our findings of new **KET** metabolites provide important new information that builds the foundation for future neurological activity studies of this important drug for the treatment of neurological diseases for diverse research fields in neuroscience and drug metabolism.

## Methods

### Chemicals

Solvents and reagents were purchased from Sigma-Aldrich or Fisher Scientific and were used without further purification. Authentic standards were also purchased from Sigma-Aldrich, Merck, or Fisher Scientific, including **(2*****R*****,6*****R*****)-hydroxy-nKET** hydrochloride metabolite. LC–MS-grade solvents were used for LC-ESI-MS analysis.

### Animal Experiments

All experimental procedures were performed according to ethical approval by the Malmö–Lund ethical Committee on Animal Research (Dnr 5.8.18-05527/19) and conducted according to the CODEX guidelines by the Swedish Research Council, Directive 2010/63/EU of the European Parliament on the protection of animals used for scientific purposes, and Regulation (EU) 2019/1010 on the alignment of reporting obligations. This study complies with ARRIVE (Animal Research: Reporting In Vivo Experiments) guidelines for reporting of animal experiments. Experiments were carried out on 9 adult male pigs (*Sus scrofa domesticus*/Danish landrace) ranging from 45 to 50 kg. Anesthetic doses of **KET** (5 mg/kg/min), fentanyl (2.5 μg/kg/min), and midazolam (0.25 mg/kg/min) were administered in saline drip through an intravenous catheter for the duration of surgery, which lasted approximately 60 min per animal. Skin and subcutaneous tissue was resected with scalpels and neck muscles were parted through blunt dissection with forceps. The sheath containing the common carotid artery (CCA), vagus nerve and internal jugular vein (IJV) could be found deep to the thymus gland. Each of the two vessels and nerve were separated using blunt dissection. Sampling from the IJV and CCA was achieved by running a single suture in order to elevate the vessels upon which a 22G cannula was inserted and approximately 10 mL of blood sampled. Blood was transferred directly to anticoagulant vacutainers and stored on ice. Plasma was separated in each blood sample using protocol sent previously. Pigs were euthanized by intravenous injection with pentobarbital. Directly after death, approximately 5 min, CSF was sampled from the cisterna magna. CSF was additionally centrifuged to remove as much blood contaminant as possible and stored on ice. Whole pig brains were extracted approximately 15 min after death and immediately frozen in liquid nitrogen, along with CSF and plasma samples.

### Data Analysis and Structural Validation

The chromatograms and mass spectra were processed using the XCMS R package for peak alignment and retention time correction, in both positive and negative ionization mode.^24^ Simultaneously, a tentative identification was automatically performed via the human metabolome database based on the high mass accuracy (threshold of 10 ppm).^25^ Features with assigned common names including the terms “ketamine” were initially used as a guide for further selection. Subsequently, known metabolites from the literature were manually added in the list.^8,12,26,27^ This included both phase I and II metabolites. The chromatograms acquired from all the QC types were thoroughly examined and tandem MS (MS/MS) were acquired in pooled biofluid samples in positive or negative ionization mode with CID of 20 eV, depending on the analyte. The derived product ion spectra were compared with already published validated tandem spectra.^8,12,26,27^ In addition, MS/MS spectra were collected from the reference standard **(2*****R*****,6*****R*****)-hydroxy-nKET** for comparison of the retention time and the specific product ions with the acquired data. The stability and performance of the experimental set over time was evaluated by plotting the intensities of the included internal standards and the QC samples were plotted against the UPLC-MS/MS sample injection order.^28^

## Supporting Information Available

Supporting figures and tables detailing structure elucidation of KET metabolites, brain distribution of these metabolites, additional experimental details, and experimental details on all investigated KET metabolites (PDF)

## ABBREVIATIONS

CCA: common carotid artery
CSF: cerebrospinal fluid
IJV: internal jugular vein
CTX-F: frontal cortex
CTX-O: occipital cortex
CTX-P: parietal cortex
CTX-T: temporal cortex
HC: hippocampus
STRIA: striatum
MDB: midbrain
OB: olfactory bulb
CBL: cerebellum
WM: white matter
PITUI: pituitary gland
CP: choroid plexus
KET: ketamine
nKET: norketamine
Gluc: glucuronide
hKET: hydroxy-ketamine
hKETa: isomer of hydroxy-ketamine
MS: mass spectrometry
MS/MS: tandem MS
QC: quality control
ACN: acetonitrile
UHPLC: ultrahigh performance liquid chromatography
IS: internal standard;
