# Supplementary material for: Identification of New Ketamine Metabolites and Their Detailed Distribution in the Mammalian Brain
Source: ACS Chem Neurosci. 2024 Mar 20;15(7):1335–41. doi: 10.1021/acschemneuro.4c00051 (PMC10995950; doi:10.1021/acschemneuro.4c00051)
Supplement: Supplementary file 1 — cn4c00051_si_001.pdf [file cn4c00051_si_001.pdf]

SUPPORTING INFORMATION

Identification of new ketamine metabolites and their detailed distribution in the mammalian brain

Theodosia Vallianatou[§], Carina de Souza Anselmo[§], Ioanna Tsiara[§], Nicholas B. Bechet[†,‡], Iben Lundgaard[†,‡], Daniel Globisch[§,*]

[§] Department of Chemistry-BMC, Science for Life Laboratory, Uppsala University, Box 576, 75123 Uppsala, Sweden

[†] Department of Experimental Medical Science, Lund University, 22362 Lund, Sweden

‡ Wallenberg Centre for Molecular Medicine, Lund University, 22362 Lund, Sweden

Corresponding author email address: Daniel.globisch@kemi.uu.se

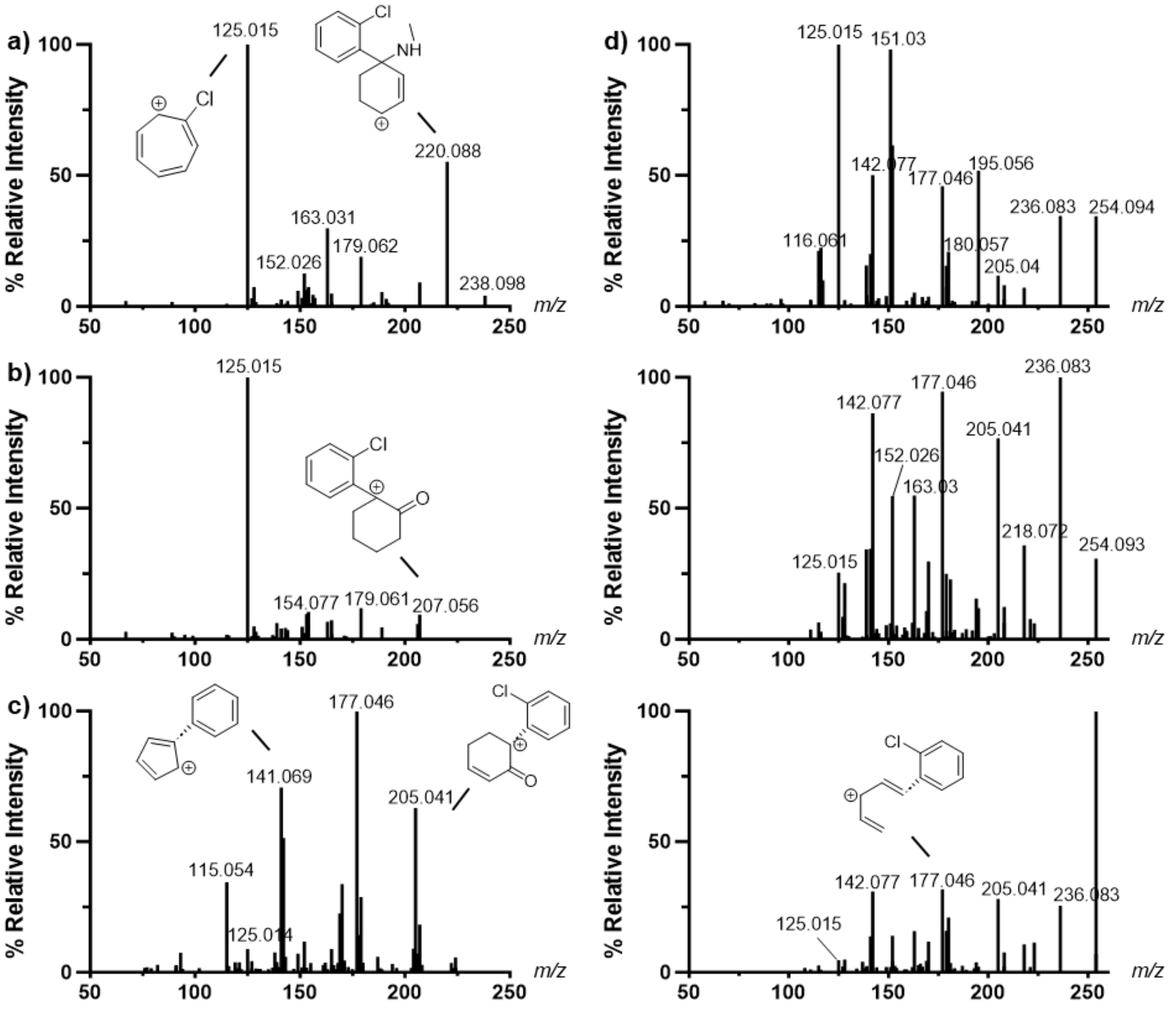

Figure S1. Structure validation of KET and its metabolites. **a)** Tandem MS spectra collected in biofluid samples for **KET** (precursor *m/z* 238.098); **b)** Tandem MS spectra collected in biofluid samples for **nKET** (precursor *m/z* 224.084); **c)** Tandem MS spectra collected in biofluid samples for **5,6-dehydro-nKET** (precursor *m/z* 222.068); **d)** Tandem MS spectra collected in biofluid samples for three **hydroxy-KET** isomers (precursor *m/z* 254.094) eluted at different retention times. Tandem spectra were collected with collision energy of 20V in positive ionization mode. Presented tandem MS spectra include *m/z* peaks with % relative intensities >1 and some obtained MS fragments are annotated.

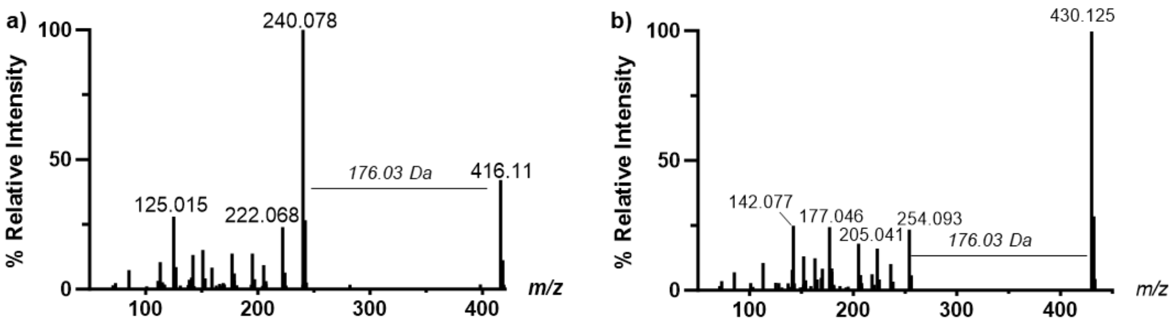

Figure S2. Structure validation of glucuronidated KET metabolites. **a)** Tandem MS spectra collected in biofluid samples for **hydroxy-nKET-Gluc** (precursor *m/z* 416.111, 5.71 min); **b)** Tandem MS spectra collected in biofluid samples for **hydroxy-KET-Gluc** (precursor *m/z* 430.126, 7.32 min). Tandem spectra were collected with collision energy of 20V in positive ionization mode. Presented tandem MS spectra include *m/z* peaks with % relative intensities >1.

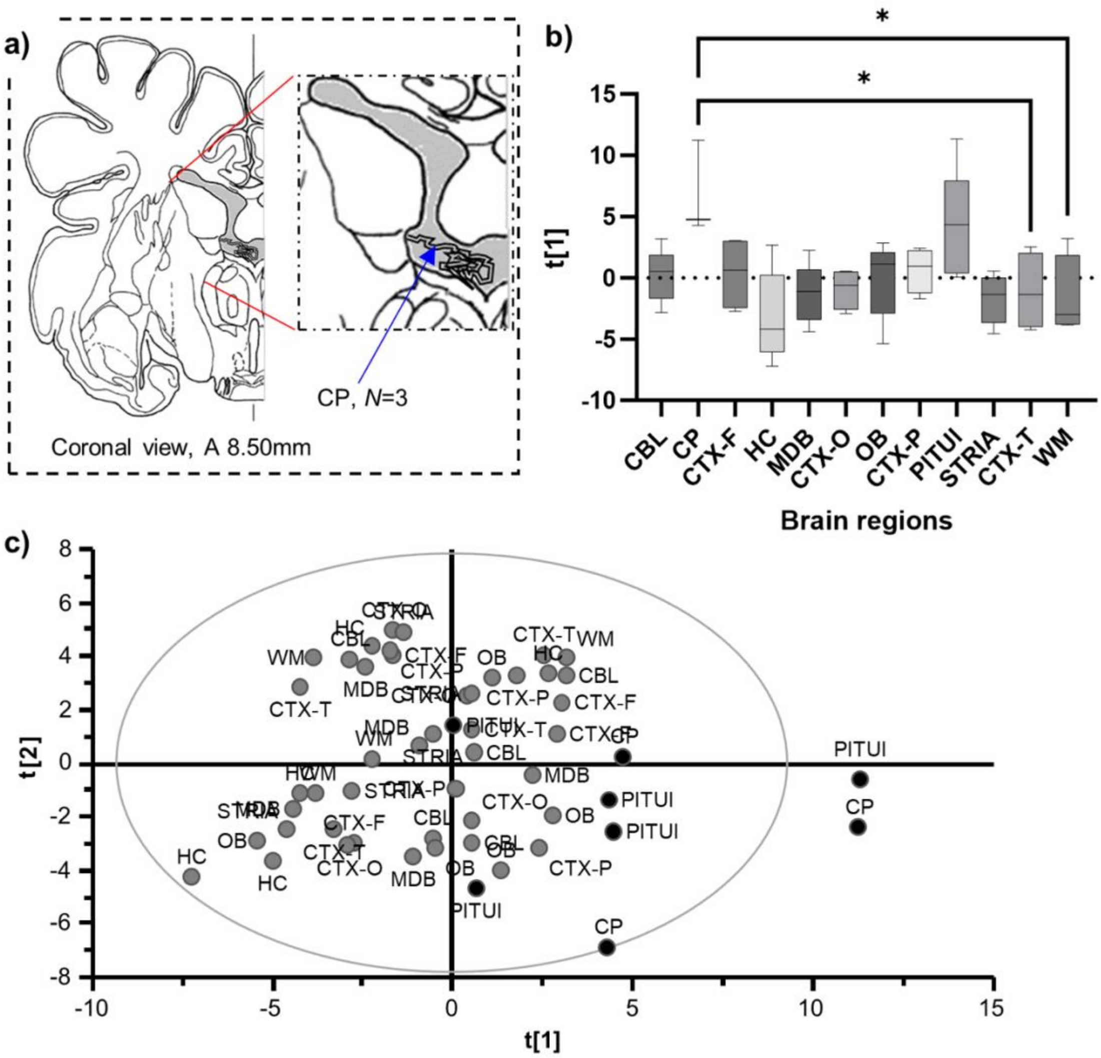

Figure S3. Brain mapping of ketamine metabolism. a) Graphical illustration of the choroid plexus (CP) located in the lateral ventricles of the pig brain; **b**) Box plot of the first principal component scores values ([t1]) for each brain region (N= 5-6 per region, N= 3 for CP) derived from the PCA including ketamine and its metabolites. **c**) Scores plot of the two first principal components deriving from the PCA on the brain regional data. $^{**}P<0.01$, $^{*}P<0.05$ (one-way mixed-effects ANOVA).

Table S1. Phase I and phase II ketamine (KET) metabolites observed in the pig brain.

Metabolite	Molecular Formula	Exact $[M+H]^+$	RT (min)	Confidence level*
5,6-dehydro-nKET	$C_{12}H_{12}ClNO$	222.0686	8.08	2a
nKET	$C_{12}H_{14}ClNO$	224.0842	8.80	2a
5,6-dehydro-nKET-r			12.08	2b
OH-5,6-dehydro-nKET	$C_{12}H_{12}ClNO_2$	238.0635	11.08	2b
KET	$C_{13}H_{16}ClNO$	238.0999	8.75	2a
hydroxy-nKET	$C_{12}H_{14}ClNO_2$	240.0791	6.36	3
(2*R*,6*R*)-hydroxy-nKET			6.66	1
hydroxy-nKET			7.23	2a
hydroxy-nKET			7.54	2a
hydroxy-nKET			8.46	2a
hydroxy-KET	$C_{13}H_{16}ClNO_2$	254.0948	6.80	2a
hydroxy-KET			7.37	2a
hydroxy-KET			7.62	2a
hydroxy-KET a (hKETa)			11.81	2a
phenol-hydroxy-nKET	$C_{12}H_{14}ClNO_3$	256.0740	6.66	2b
dihydroxy-nKET			7.80	2b
M2 (Glucuronidated-$C_{12}H_{14}ClNO$)	$C_{18}H_{22}ClNO_7$	400.1163	9.16	3
OH-5,6-dehydro-nKET-Gluc	$C_{18}H_{20}ClNO_8$	414.0956	9.27	2b
hydroxy-nKET-Gluc	$C_{18}H_{22}ClNO_8$	416.1112	5.42	2a
hydroxy-nKET-Gluc			6.56	2a
hydroxy-nKET-Gluc			5.71	2a
hydroxy-nKET-Gluc			7.31	2a
hydroxy-nKET-Gluc			8.30	2a
hydroxy-nKET-Gluc			11.63	2a
hydroxy-nKET-Gluc			11.91	2a
hydroxy-KET-Gluc	$C_{19}H_{24}ClNO_8$	430.1269	5.73	2a
hydroxy-KET-Gluc			5.92	2a
hydroxy-KET-Gluc			7.32	2a
hydroxy-KET-Gluc			12.03	2a
hydroxy-KET-Gluc			12.24	2a
M1 (Glucuronidated-$C_{12}H_{14}ClNO_3$)	$C_{18}H_{22}ClNO_9$	432.1061	5.29	4

* The highest validation level of detected metabolite structures was obtained with the commercially available reference compound **(2*R*,6*R*)-hydroxy-nKET** (Confidence level 1). The structures of **KET**, **nKET**, **5,6 dehydro-nKET**, and hydroxylated derivatives were validated by comparison of the MS/MS acquired with MS/MS spectra from experimental or computational libraries with either HMDB, the software SIRIUS or literature spectra (Confidence level 2a (library) and Confidence level 2b (experimental). The metabolites for which we had indication of the molecular formula with MS/MS spectrum but without structural elucidation were identified as Confidence level 3. The metabolites that we only had indication of the molecular formula without an MS/MS spectrum to confirm were identified at Confidence level 4.

Table S2. Mean intensities (a.u.) of the detected metabolites in all biofluids. SEM, standard error of the mean

Metabolite	CCA		CSF		IJV	
	**Mean**	**SEM**	**Mean**	**SEM**	**Mean**	**SEM**
5,6-dehydro-nKET	2.60E+06	3.69E+05	5.47E+05	9.73E+04	2.00E+06	3.69E+05
nKET	1.55E+07	2.55E+06	5.88E+06	1.54E+06	1.28E+07	2.43E+06
5,6-dehydro-nKET-r	1.10E+05	2.07E+04	6.95E+04	1.66E+04	8.69E+04	2.10E+04
OH-5,6-dehydro-nKET	1.23E+06	2.00E+05	8.19E+05	3.51E+05	8.90E+05	1.87E+05
KET	1.27E+08	8.00E+06	9.44E+07	9.80E+06	1.09E+08	1.07E+07
hydroxy-nKET (6.36 min)	1.41E+05	2.85E+04	4.65E+04	8.97E+03	1.15E+05	2.58E+04
(2R,6R)-hydroxy-nKET	2.04E+07	3.43E+06	1.36E+07	2.86E+06	1.70E+07	3.63E+06
hydroxy-nKET (7.23 min)	2.35E+06	5.79E+05	7.95E+05	1.87E+05	2.21E+06	6.56E+05
hydroxy-nKET (7.54 min)	8.01E+05	2.08E+05	7.19E+05	1.56E+05	8.05E+05	1.94E+05
hydroxy-nKET (8.46 min)	6.92E+06	1.71E+06	2.90E+06	7.45E+05	5.22E+06	1.56E+06
hydroxy-nKET (7.23 min)	3.13E+06	6.24E+05	1.65E+06	3.95E+05	2.41E+06	5.41E+05
hydroxy-nKET (7.54 min)	3.42E+06	6.30E+05	1.76E+06	4.56E+05	2.65E+06	6.19E+05
hydroxy-nKET (8.46 min)	2.78E+06	6.22E+05	2.17E+06	5.32E+05	2.38E+06	6.72E+05
hydroxy-KET (6.80 min)	1.00E+05	2.42E+04	3.96E+05	1.53E+05	1.13E+05	2.63E+04
phenol-hydroxy-nKET	6.83E+05	1.26E+05	3.52E+04	8.27E+03	5.83E+05	1.37E+05
dihydroxy-nKET	1.33E+06	2.19E+05	1.17E+05	2.16E+04	1.16E+06	2.34E+05
OH-5,6-dehydro-nKET-Gluc	3.00E+05	5.57E+04	4.06E+03	5.03E+02	2.67E+05	4.68E+04
hydroxy-nKET-Gluc (5.42 min)	6.68E+05	1.52E+05	2.01E+04	4.80E+03	5.75E+05	1.53E+05
hydroxy-nKET-Gluc (6.56 min)	3.04E+07	4.69E+06	3.02E+05	1.16E+05	2.48E+07	4.79E+06
hydroxy-nKET-Gluc (5.71 min)	1.22E+06	2.71E+05	1.65E+04	7.60E+03	6.98E+05	1.59E+05
hydroxy-nKET-Gluc (7.31 min)	3.18E+06	5.01E+05	2.59E+04	1.04E+04	2.83E+06	4.97E+05
hydroxy-nKET-Gluc (8.30 min)	4.67E+05	9.66E+04	5.62E+03	1.76E+03	3.41E+05	7.75E+04
hydroxy-nKET-Gluc (11.63 min)	2.45E+06	5.45E+05	2.08E+04	7.19E+03	2.10E+06	4.79E+05
hydroxy-nKET-Gluc (11.91 min)	2.16E+06	6.33E+05	2.18E+04	7.81E+03	1.65E+06	4.47E+05
hydroxy-KET-Gluc (5.73 min)	3.95E+05	9.36E+04	1.40E+04	3.17E+03	3.27E+05	9.10E+04
hydroxy-KET-Gluc (5.92 min)	3.35E+06	6.03E+05	2.51E+04	1.11E+04	2.73E+06	6.15E+05
hydroxy-KET-Gluc (7.32 min)	1.92E+07	3.11E+06	1.05E+05	4.49E+04	1.72E+07	3.09E+06
hydroxy-KET-Gluc (12.03 min)	1.50E+06	4.23E+05	1.11E+04	2.74E+03	1.05E+06	3.30E+05
hydroxy-KET-Gluc (12.24 min)	2.03E+06	5.28E+05	2.46E+04	5.67E+03	1.53E+06	5.14E+05
M1	3.59E+05	8.31E+04	3.75E+03	7.31E+02	3.94E+05	1.17E+05

Experimental part

Tissue processing and sample preparation.

The preparation of the plasma (CCA, IJV) and CSF samples was performed simultaneously. An aliquot of 100 μL was collected from each sample (N=9 per sample type). As internal standard (I.S.), a mixture of C-13 isotopically labeled tyrosine (5 μg/mL), phenylalanine (10 μg/mL) and valine (30 μg/mL) were added to every sample at a volume of 20 μL. Sample preparation was performed in ice.

Quality control (QC) samples were prepared by 10 μL aliquots from all samples (QC_CCA, QC_CSF, QC_IJV). LC-MS grade MeOH (480 μL; a ratio of 1:4 of sample: MeOH) was added and the mixture was vortexed before being cooled under -20 °C for 1 hour. The mixture was then centrifuged (5 min, 18,620 g, 4 °C) and the supernatant isolated. Lyophilisation in speed vac (V-AL mode) followed (until dry residue). The samples were stored in -20 °C until the UPLC-MS analysis. Samples were reconstituted in 100 μL H_2O:ACN (95:5) and centrifuged again (5 min, 18,620 g, 4 °C). The supernatant was transferred to LC-MS vials. One additional QC sample was prepared by 5 μL aliquots of each sample (excluding the already prepared fluid-specific QCs).

The brain regions were weighed and transferred into beads-containing vials. Methanol:H_2O (80:20) was added approximately at a concentration of 4 μL/mg brain tissue to every sample. As internal standard (I.S.), a mixture of C-13 isotopically labeled tyrosine (5 μg/mL), phenylalanine (10 μg/mL) and valine (30 μg/mL). The volume of the I.S. mixture added to every sample was adjusted according to the corresponding sample weight. The homogenization was performed in a Lysing matrix D instrumentation (MP Biomedicals) in dry ice at a cycle of 20 s shaking (4 m/s) and 30 s performed three times.17 Samples were collected, precipitated in ice for 1 h and centrifuged at 13,400 rpm for 5 min. The supernatant was collected and dried under vacuum on a Speedvac and subsequently stored in -20 °C for a maximum of three days prior to analysis. Samples were re-suspended with water:acetonitrile (95:5) prior to UPLC-MS/MS analysis, at a volume normalized to the sample weight. Quality control (QC) samples were prepared by 5 μL aliquots from all samples.

U(H)PLC-mass spectrometry.

The UPLC-MS/MS analysis was performed in either a SYNAPT G2-S High Definition Mass Spectrometer (Waters) or a Maxis II ETD Q-Tof mass spectrometer (Bruker) using an electrospray ionization (ESI) source with either an AQCUITY UPLC I-class system or an Elute UHPLC system, respectively, and equipped with a Waters ACQUITY UPLC® HSS T3 column (1.8 μm, 100 × 2.1 mm). Water with 0.1% formic acid was used as mobile phase A and methanol with 0.1% formic acid was used as mobile phase B. The column temperature was kept at 40 °C, and the autosampler at 6 °C. The flow rate was set to 0.2 mL/min. The gradient used was as follows: 0–2 min, 0% B; 2–15 min, 0-100% B; 15–16 min,100% B; 16–17 min, 100–0% B; 17–21 min, 0% B.

The system was controlled either using the MassLynx software package v 4.1 (Waters) or using the Compass HyStar software package (Bruker). High-resolution mass spectra were acquired in positive and negative ionization mode, at a mass range of *m/z* 50-1500. Data acquisition in the SYNAPT G2-S HDMS was performed in MSE mode. The samples were injected to the UPLC-MS system in a randomized order with QC samples injected in the beginning and end of the sample list in both ionization modes, as well as after every eight samples (7 QCs in each ionization mode in total). The structural validation of the anesthetic drugs was carried out in a Maxis II ETD Q-TOF mass spectrometer (Bruker Daltonics, Germany) using an electrospray ionization (ESI) source with an Elute UHPLC (Bruker Daltonics, Germany) system. The separation was performed on an Acquity UPLC HSS T3 column (1.8 μm, 100 × 2.1 mm) from Waters Corporation. The mobile phase A was constituted of Milli-Q water and the mobile phase B of methanol, both with 0.1% formic acid. The column temperature was kept at 40 °C, and the autosampler temperature at 4 °C. The flow rate was set to 0.22 mL/min with an injection volume of 5 μL. The LC gradient used was as follows: 0–2 min, 0% B; 2–15 min, 0–100% B; 15–16 min, 100% B; 16–17 min, 100–0% B; 17–23 min, 0% B. The system was controlled using the Compass HyStar software package from Bruker (Bruker Daltonics, Germany). High-resolution mass spectra were acquired in both positive and negative modes at a mass range of *m/z* 50–1200. Data acquisition was performed in AutoMSMS mode (data-dependent acquisition, DDA) with a cycle time of 0.5 s and a ramped collision energy from 20 to 50 eV. At the beginning of each run, a solution of sodium formate [10 mM in a mixture of 2-propanol/water (1/1, v/v)] was used for internal calibration, in a segment between 0.10 and 0.31 min
